# Proton Therapy in Supradiaphragmatic Lymphoma: Predicting Treatment-Related Mortality to Help Optimize Patient Selection

**DOI:** 10.1016/j.ijrobp.2021.10.151

**Published:** 2022-03-15

**Authors:** Georgios Ntentas, Katerina Dedeckova, Michal Andrlik, Marianne C. Aznar, Rebecca Shakir, Johanna Ramroth, Rubina Begum, Jiří Kubeš, Sarah C. Darby, N. George Mikhaeel, David J. Cutter

**Affiliations:** ⁎ Nuffield Department of Population Health, University of Oxford, Oxford, United Kingdom; † Guy's and St Thomas’ NHS Foundation Trust, Department of Medical Physics, London, United Kingdom; ‡ Proton Therapy Center Czech, Prague, Czech Republic; § Department of Oncology, 2nd Faculty of Medicine Charles University in Prague and Motol University Hospital, Prague, Czech Republic; ║ Manchester Cancer Research Centre, University of Manchester, Manchester, United Kingdom; ¶ Oxford Cancer Centre, Oxford University Hospitals NHS Foundation Trust, Oxford, United Kingdom; # Guy's & St Thomas’ NHS Foundation Trust and School of Cancer & Pharmaceutical Sciences, King's College London, London, United Kingdom

## Abstract

**Purpose:**

In some patients with Hodgkin lymphoma (HL), proton beam therapy (PBT) may reduce the risk of radiation-related cardiovascular disease (CVD) and second cancers (SC) compared with photon radiation therapy (RT). Our aim was to identify patients who benefit the most from PBT in terms of predicted 30-year absolute mortality risks (AMR_30_) from CVD and SC, taking into account individual background, chemotherapy, radiation, and smoking-related risks.

**Methods and Materials:**

Eighty patients with supradiaphragmatic HL treated with PBT between 2015 and 2019 were replanned using optimal photon RT. To identify patients predicted to derive the greatest benefit from PBT compared with photon RT, doses and AMR_30_ from CVD and SC of the lung, breast, and esophagus were compared for all patients and across patient subgroups.

**Results:**

For patients with mediastinal disease below the origin of the left main coronary artery (n = 66; 82%), PBT reduced the mean dose to the heart, left ventricle, and heart valves by 1.0, 2.7, and 3.6 Gy, respectively. Based on U.S. mortality rates, PBT reduced CVD AMR_30_ by 0.2%, from 5.9% to 5.7%. The benefit was larger if the mediastinal disease overlapped longitudinally with the heart by ≥40% (n = 23; 29%). PBT reduced the mean dose to the heart, left ventricle, and heart valves by 3.2, 5.6, and 5.1 Gy, respectively, and reduced CVD AMR_30_ by 0.8%, from 7.0% to 6.2%. For patients with axillary disease (n = 25; 31%), PBT reduced the mean lung dose by 2.8 Gy and lung cancer AMR_30_ by 0.6%, from 2.7% to 2.1%. Breast and esophageal doses were also lower with PBT, but the effects on AMR_30_ were negligible. The effect of smoking on CVD and lung cancer AMR_30_ was much larger than radiation and chemotherapy and the differences between radiation modalities.

**Conclusions:**

The predicted benefit of PBT is not universal and limited to certain categories of patients with lymphoma and lower mediastinal or axillary disease. Smoking cessation should be strongly encouraged in smokers who require thoracic RT.

## Introduction

Patients with Hodgkin lymphoma (HL) are at risk of treatment-related long-term effects, including cardiovascular disease (CVD) and second cancers (SC) of the lung, breast, and esophagus.^1^, ^2^, ^3^, ^4^, ^5^, ^6^, ^7^ Most estimates of the magnitude of these risks are based on patients treated many decades ago using outdated radiation therapy (RT) techniques (eg, mantle RT, which delivered high radiation doses to target volumes and organs at risk [OARs]). In recent decades, reductions in treatment volumes and prescription doses, as well as advances in medical imaging and photon RT technology, have maintained lymphoma treatment efficacy while reducing incidental radiation doses to OARs substantially.^8^, ^9^, ^10^, ^11^ As a result, some studies have reported that patients treated in the last few decades have lower risks than those treated many decades ago.^12^, ^13^, ^14^, ^15^ However, even for those treated today, the risks of CVD and SC in some patients with HL may remain higher than those of the general population.

The introduction of proton beam therapy (PBT), which can deliver radiation doses more conformally than conventional RT, has offered the potential to further reduce radiation doses to OARs when treating patients with HL.^16^, ^17^, ^18^ However, PBT is more complex and expensive than conventional RT and less widely available, raising the need for appropriate patient selection. Recent guidelines by the International Lymphoma Radiation Oncology Group (ILROG)^19^ identify subgroups of patients with HL who may benefit substantially from PBT compared with photon RT. These subgroups include patients whose mediastinal disease extends below the origin of the left main stem coronary artery (LMSCA), young women, and patients with relapsed disease.

Reducing radiation doses to OARs is expected to be beneficial, but the link between dose reduction and the absolute magnitude of clinical benefit is not always clear. Late effects depend on other factors, as well as radiation dose, including age, sex, chemotherapy use, smoking status, and the disease rates of the general population in the patient's geographic region. Therefore, considering radiation dose reduction alone does not give a full picture of the expected clinical benefit of PBT. The purpose of this study is to compare PBT with the current best photon RT technique to identify which patients benefit the most from PBT, not only in terms of radiation dose reductions to specific OARs, but by predicting the more clinically meaningful endpoint of 30-year absolute mortality risk (AMR_30_) for CVD and SC in a large group of patients with HL who were treated with PBT. In addition to radiation-related risk, we also examined the role of other risk factors in determining AMR_30_, namely chemotherapy, smoking status, and mortality rates in the general population. To our knowledge, this is the first study to evaluate the selection of patients with lymphoma for PBT using AMR_30_ and clinical data from patients actually treated with PBT.

## Methods and Materials

### Patients and treatment planning

Between April 2015 and April 2019, a total of 80 consecutive patients with HL and mediastinal disease (50 women and 30 men) were treated with anthracycline-containing chemotherapy, followed by pencil beam scanning PBT in deep inspiration breath hold (DIBH) at the Proton Therapy Center Czech s.r.o. The prescribed doses in Gy equivalent (GyE; relative biologic effectiveness of 1.1) were 30 GyE in 15 fractions (75 patients), 20 GyE in 10 fractions (3 patients), 36 GyE in 18 fractions (1 patient), and 40 GyE in 20 fractions (1 patient). Radiation target volumes were defined as involved sites for all early and intermediate-stage patients and residual disease for advanced-stage patients.

The ILROG recommendations for gross tumor volume and clinical target volume (CTV) definitions were used.^8^ The heart and cardiac substructures were contoured according to published atlases.^20^^,^^21^ The PBT planning methodologies have been described in a previous study.^17^ Alternative photon RT plans were produced using the same DIBH scans. CTV to planning target volume (PTV) margins that reflect current clinical practice were used (ie, 5 mm for photon RT plans^11^^,^^22^ and 7-12 mm for PBT plans).^17^ For both proton and photon therapy, OAR dose objectives were set individually to achieve the lowest possible doses considering the individual anatomy and clinical characteristics for the patient.

All plans were produced by experienced physicists, and reviewed and approved by senior radiation oncologists from 2 different institutions (University of Oxford and Guy's and St Thomas’ NHS Foundation Trust). The photon RT planning method used for most patients with mediastinal and neck disease was butterfly volumetric arc therapy (BVMAT),^11^ but for more complex volumes (eg, those involving the axilla or extended volumes), additional plans were produced using BVMAT with additional partial arcs (PartArc)^17^ or full-arc BVMAT (FaB-VMAT)^23^ and then compared. In total, 3 planning methods were used: BVMAT (45 patients), PartArc (30 patients), and FaB-VMAT (5 patients) depending on the photon RT deemed the best plan based on PTV coverage and OAR doses. All plans were produced on Eclipse, version 15.0. The study was approved by the institutional review board of the Proton Therapy Center Czech s.r.o.

### Comparison of radiation doses

For each individual, the difference between photon RT and PBT was calculated for the radiation dose to the OAR (whole heart, 12 cardiac substructures, lungs, breast, esophagus, larynx, spinal cord, and thyroid), RT PTV, and normal tissue dose (whole body minus PTV). To identify patients who would derive the greatest benefit from PBT in dosimetric terms, cardiac dose differences were compared separately for patients whose mediastinal disease extended above or below the origin of the LMSCA, as suggested in the ILROG guidelines,^19^ and for patients whose disease extended above or below the 7th thoracic vertebra (T7).^17^

Additionally, the percentage longitudinal overlap between the extent of the disease (specified by CTV extent) and the heart (Appendix A-Fig. E1) was examined as a potential indicator of benefit from PBT for heart doses. Lung and breast doses were also studied for patients with and without axillary disease. The differences in doses between these subgroups and by sex were examined using a linear regression model. Significance tests were 2-sided and conducted using the *t* distribution. Calculations were performed in Stata, version 14.2 (Stata Corp LLC).

### Cardiovascular and second cancer risk prediction

The AMR_30_ for CVD and for each of the SC was predicted for the patients in this study, taking into account the competing risk of death from other causes, and calculated in 3 steps. First, for each of the 80 patients, the background cumulative mortality risk (in the absence of any lymphoma- or treatment-related risk) was predicted using mortality rates in the general population. To do this, attained-age and sex-specific mortality rates corresponding to each patient were obtained from the World Health Organization mortality database.^24^ Then, these risks were averaged across all 80 patients for each of the 30 years after treatment. Second, the calculations were repeated taking into account the chemotherapy-related increase in the mortality rate for CVD.^3^ Finally, the calculations were repeated again taking into account the RT-related increases in CVD and SC mortality rates, as well as the chemotherapy-related increase in CVD.

The above treatment-related increases in the mortality rates for CVD and SC were derived from published dose–response relationships, including age at treatment-specific values where appropriate combined with patient-specific mean radiation doses to relevant organs or cardiac substructures (Table E5). For CVD, the mortality rate, including the effect of treatment, was the sum of the mortality rates for coronary heart disease,^2^ congestive heart failure,^3^ valvular heart disease,^1^ other cardiac diseases, and stroke.^25^ Dose–response relationships were not available for other cardiac diseases (eg, pericardial disease and arrhythmias), and the approach used for this group is described in Appendix B.

Risks were predicted for all patients combined, as well as for the patient subgroups in the dosimetric analysis. Separate calculations were performed using background mortality rates in the 4 different geographic regions in which PBT is currently available (United States, Western Europe, Eastern Europe, and Japan). Finally, U.S. mortality rates were used to investigate the effect of smoking on the cumulative mortality risks for CVD and lung cancer for 30-year-old male and female current smokers and never-smokers by assuming the average mean heart dose (MHD), mean left ventricular dose (MLVD), and heart valve dose (SumValve) delivered by PBT and photon RT in patients with ≥40% CTV-to-heart overlap for CVD. For lung cancer, the average mean lung dose (MLD) received by patients with axillary disease was used. The risk prediction methodology is detailed in Appendices B and C.

## Results

### Characteristics of patients with HL

The median age at the time of treatment was 30 years (range, 18-79 years), and 50 patients (62%) were female (Table 1). Sixty-seven patients (84%) had early favorable or intermediate (IIA/B) stage disease. All patients completed PBT without interruptions. With a median follow-up time of 24 months (range, 3-56 months), all but 2 patients (97.5%) achieved complete remission. No severe (grade 3-4) toxicities or radiation pneumonitis of any grade were reported. Grade 2 toxicities were reported for a few patients (Table E4).

**Table 1:** Characteristics of 80 patients with supradiaphragmatic Hodgkin lymphoma treated with proton beam therapy

	Number of patients	(%)
**Sex**		
Male	30	(38)
Female	50	(62)
**Age at time of RT**		
Median age: 30.5 y (range, 18-79 y)	―	―
**Smoking status at time of HL diagnosis**		
Never smoker	39	(49)
Current/former smoker	25	(31)
Unknown	16	(20)
**Follow up after RT**		
Median follow up: 24 mo (range, 3-56 mo)	―	―
HL classification*		
Early	3	(4)
Intermediate	64	(80)
Advanced	12	(15)
Relapse	1	(1)
**Disease sites involved**		
Mediastinum only	20	(25)
Mediastinum and left neck	9	(11)
Mediastinum and right neck	1	(1)
Mediastinum and bilateral neck	21	(26)
Mediastinum, neck, and left axilla	10	(13)
Mediastinum, neck, and right axilla	16	(20)
Mediastinum, neck, and bilateral axilla	3	(4)
**RT CTV**		
Median volume: 598.0 cc (range, 82-1732 cc)		
**RT technique used for photon RT replans**		
BVMAT	45	(56)
PartArc	30	(38)
FaB-VMAT	5	(6)
**Chemotherapy**		
Median dose of anthracyclines 340 mg/m^2^ (range, 170-420 mg/m^2^)		
6 × escalated BEACOPP	15	(23)
2 × escalated BEACOPP + 2 × ABVD	37	(51)
4 × ABVD	16	(21)
Other	12	(5)
**Patient subgroups**		
**Longitudinal overlap CTV/heart**		
<40% overlap	57	(71)
≥40% overlap	23	(29)
**CTV inferior extension vs left main stem coronary artery**		
At and above only	14	(18)
Below	66	(82)
**CTV inferior extension vs vertebral thoracic level**		
At and above seventh thoracic level	33	(41)
Below seventh thoracic level	47	(59)
**Axillary involvement**		
No	55	(64)
Yes	29	(36)
**Total number of patients**	80	(100)

*Abbreviations*: ABVD = adriamycin, bleomycin, vinblastine, and dacarbazine; BEACOPP = bleomycin, etoposide, doxorubicin, cyclophosphamide, vincristine, procarbazine, and prednisolone; BVMAT = butterfly volumetric arc therapy; CTV = clinical target volume; FaB-VMAT = BVMAT with an additional full-arc; HL = Hodgkin lymphoma; PartArc = BVMAT with additional partial arcs; RT = radiation therapy.

⁎ German Hodgkin Study Group's risk classification system.

### Comparison of radiation doses from PBT and photon-RT

For all 80 patients, PTV coverage was >95% of the prescribed dose for both PBT and photon RT plans. When all patients were considered together, PBT reduced the average MHD compared with photon RT but not significantly (–0.7 Gy; *P* = .06). However, PBT delivered significantly lower average MLVD (–2.2 Gy; *P* < .001) and SumValve (–2.7 Gy; *P* < .001; Table 2). For the 82% of patients whose CTV extended below the LMSCA and the 59% of patients whose CTV extended below the T7 level, there were significant reductions in cardiac doses with PBT. For patients whose CTV did not extend below the LMSCA or T7, PBT did not confer any significant dose reductions and, in some cases, increased cardiac doses compared with photon RT (Table 2; Supplemental Table E5). The largest dose reductions for PBT compared with photon RT were observed for the approximately 30% of patients whose CTV overlapped longitudinally with the heart by *≥*40% (MHD: –3.2 Gy; MLVD: –5.6 Gy; and SumValve: –5.1 Gy; all *P* < .001; Table 2). For patients with a <40% overlap, there was no dosimetric benefit with PBT for MHD (+0.4 Gy; *P* = .29), although both MLVD and SumValve were somewhat reduced with PBT, by –0.9 Gy (*P* = .04) and –1.7 Gy (*P* = .02), respectively.

**Table 2:** Average mean organ doses from photon RT and PBT

Organ at risk; (notation for dose)	Patient group	Photon RT (Gy); average (range)	PBT (GyE*); average (range)	Absolute difference (Gy); average (range)	*P* value for absolute difference	*P* value for difference between patient subgroups
**Whole heart (mean)**	All	9.5 (0.6-26.1)	8.7 (1.0-20.7)	–0.7 (–12.5 to 5.8)	.06	―
	<40%†	6.7 (0.6-13.5)	7.1 (1.0-14.3)	+0.4 (–6.5 to 5.8)	.29	< .001
	≥40%†	16.5 (9.6-26.1)	13.3 (7.5-20.7)	–3.2 (–12.5 to 3.3)	< .001	
	Above LMSCA‡	3.0 (0.6-6.0)	4.1 (1.0-7.2)	+1.1 (0.4-2.6)	.15	.02
	Below LMSCA‡	10.9 (3.3-26.1)	9.9 (3.5-20.7)	–1.0 (–12.5 to 5.8)	< .01	
**Left Ventricle (mean)**	All	5.4 (0.2-23.9)	3.1 (0.0-14.4)	–2.2 (–18.0 to 3.9)	< .001	―
	<40%†	3.0 (0.2-9.1)	2.1 (0.0-10.0)	–0.9 (–7.5 to 2.7)	.04	< .001
	≥40%†	11.2 (2.9-23.9)	5.7 (0.1-14.4)	–5.6 (–18.0 to 3.9)	< .001	
	Above LMSCA‡	0.9 (0.2-1.38)	0.7 (0.1-2.4)	–0.2 (–1.0 to 1.2)	.82	.013
	Below LMSCA‡	6.3 (0.7-23.9)	3.6 (0.1-14.4)	–2.7 (–18.0 to 3.9)	< .001	
**Heart valves (SumValve**§**)**	All	14.1 (0.4-28.3)	11.4 (0.1-29.2)	–2.7 (–19.5 to 8.4)	< .001	―
	<40%†	11.2 (0.4-24.1)	9.5 (0.1-29.2)	–1.7 (–19.5 to 8.4)	.02	< .01
	≥40%†	21.0 (9.1-28.3)	15.9 (6.8-27.2)	–5.1 (–17.3 to 6.8)	< .001	
	Above LMSCA‡	4.5 (0.4-12.7)	6.3 (0.1-14.9)	+1.7 (–1.9 to 8.4)	.20	< .01
	Below LMSCA‡	16.1 (3.0-28.3)	12.5 (1.0-29.2)	–3.6 (–19.5 to 6.8)	< .001	
**Carotid arteries**║	All	25.1 (2.8-30.2)	27.1 (7.9-31.6)	+2.0 (–1.6 to 8.7)	< .001	―
**Lungs**	All	7.9 (3.7-14.6)	5.7 (2.4-10.1)	–2.2 (–7.1 to 0.41)	< .001	―
	No axilla	7.1 (3.7-14.1)	5.2 (2.4-9.6)	–1.9 (–4.5 to 0.41)	< .001	< .01
	Axilla	9.5 (6.5-14.6)	6.6 (3.7-10.1)	–2.8 (–7.1 to 0.16)	< .001	
**Breast**¶	All	2.5 (0.4-8.7)	1.6 (0.2-4.6)	–0.9 (–5.6-1.1)	< .001	―
	No axilla	2.0 (0.4-6.5)	1.5 (0.2-4.2)	–0.5 (–2.3 to 0.6)	.03	< .001
	Axilla	3.7 (0.7-8.7)	1.9 (0.5-4.6)	–1.8 (–6.0 to 1.1)	< .001	
**Esophagus**	All	16.4 (5.4-24.4)	15.2 (0.3-24.5)	–1.2 (–9.7 to 8.2)	< .01	―
**Total normal tissue**#	All	4.7 (1.6-8.6)	2.3 (0.7-6.2)	–2.4 (–6.4 to 0.5)	< .001	―

*Abbreviations*: CTV = clinical target volume; LMSCA = left main stem coronary artery; PBT = proton beam therapy; RT = radiation therapy.

Dose metrics for additional organs at risk and target volumes are shown in Table E5.

⁎ GyE is Gy equivalent of relative biologic effectiveness of 1.1 with PBT

† %CTV to heart longitudinal overlap was < or ≥40%

‡ CTV was above LMSCA only or extended below LMSCA

§ SumValve = (0.553 × AVMean) + (0.368 × MVMean) + (0.079 × TVMean). This is a weighted average of the mean doses to the aortic valve (AVMean), mitral valve (MVMean), and tricuspid valve (TVMean) based on data by Cutter at el.^1^

║ Average mean dose to left and right common carotid arteries

¶ Female patients only

# Mean dose to whole body minus RT planning target volume

Compared with photon RT, PBT delivered higher doses to the carotid arteries (average increase for all patients: +2.0 Gy; *P* < .001) but lower doses to the lungs (–2.2 Gy; *P* < .001), female breast (–0.9 Gy; *P* < .001), and esophagus (–1.2 Gy; *P* = .003). The reductions in lung and breast doses were greater for patients whose axilla was irradiated (lungs: –2.8 Gy; *P* < .001; breast: –1.8 Gy; *P* < .001; Table 2). Lastly, PBT halved the total normal tissue dose compared with photon RT (2.3 vs 4.7 Gy; *P* < .001; Table 2). Further details of the dosimetric comparison for additional cardiac substructures and OARs are provided in Supplemental Table E5.

### Treatment-related absolute 30-year mortality risks in different geographic regions

**Fig. 1:**
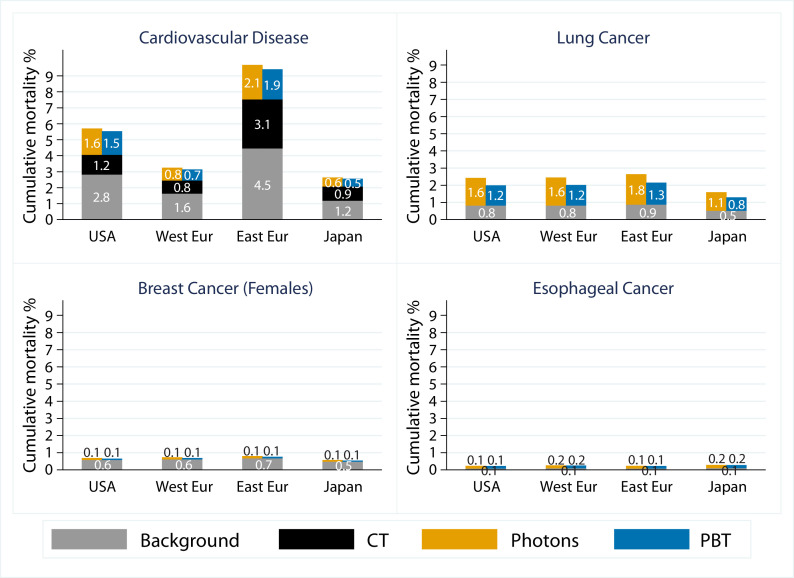
Predicted 30-year absolute mortality risk (AMR_30_) for all cardiovascular disease and second cancers. The quantities shown are the average AMR_30_ for the 80 patients in this study. AMR_30_ includes background mortality risk (gray bar), absolute excess risk after chemotherapy (black bar), absolute excess risk after photon radiation therapy (yellow bar), and proton beam therapy (blue bar). For each patient, background AMR_30_ was predicted based on their age and sex. Separate calculations were conducted using population mortality rates from each geographic region. The chemotherapy-related AMR_30_ was predicted using the excess rate ratio by Van Nimwegen et al.^3^ The radiation therapy-related AMR_30_ was predicted using the excess rate ratios, or excess relative risks, per Gy from the studies in Supplemental Table E3, combined with patient-specific radiation doses from photons and proton beam therapy. Further details are provided in Appendix B.

The background CVD AMR_30_ varied substantially between the 4 geographic regions considered. For the United States, the rate was 2.8%, which is almost double that of the Western European population (1.6%) and just over half that of the Eastern European population (4.5%). The Japanese population had the lowest CVD AMR_30_ (1.2%; Fig. 1).

**Fig. 2:**
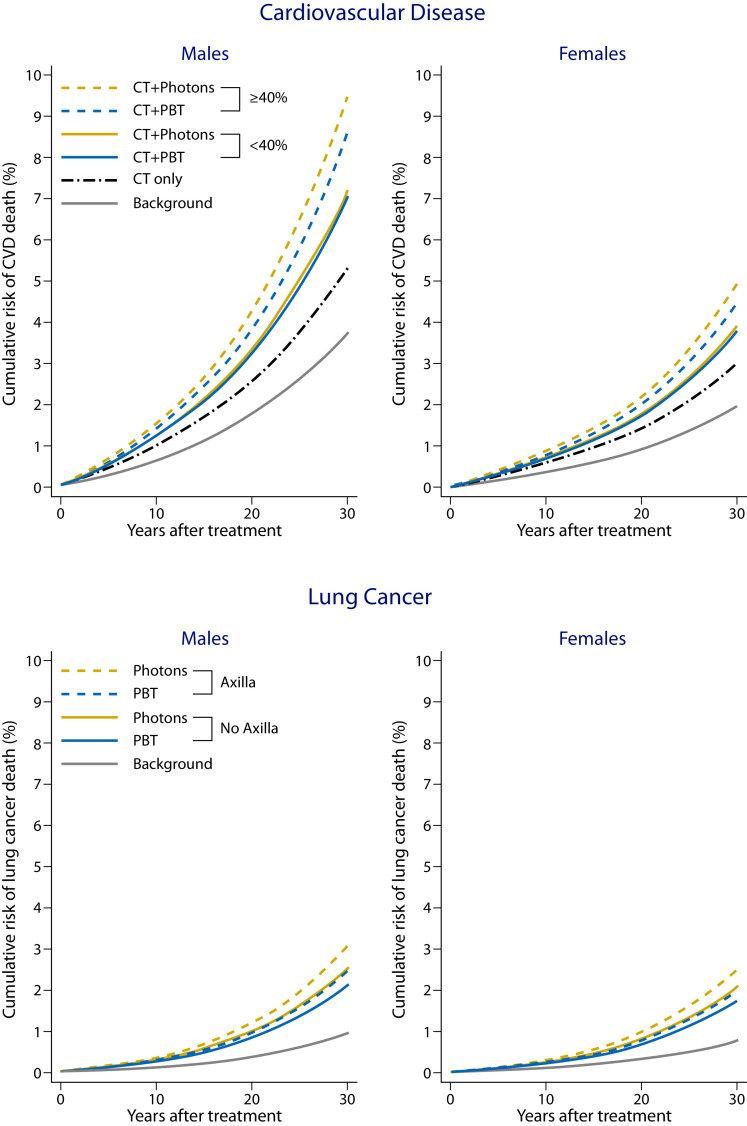
Predicted 30-year absolute mortality risk (AMR_30_) for all cardiovascular disease and lung cancer as a second primary cancer for male and female patients. Average AMR_30_ for cardiovascular disease is presented for patients with ≥40% or <40% clinical target volume to the heart longitudinal overlap and for lung cancer for patients with and without axillary disease. The background AMR_30_ was calculated using standardized U.S. mortality rates. The chemotherapy-related AMR_30_ was predicted using excess rate ratios by Van Nimwegen et al.^3^ The radiation therapy-related AMR_30_ was predicted using the excess relative risks from the studies in Supplemental Table E3, combined with the average radiation dose for each subgroup as shown in Table 2 assigned to every patient. Further details are provided in Appendix B.

The dose–response relationships suggest that both chemotherapy and RT have multiplicative effects on background mortality rates, sometimes varying with age at the time of treatment (Supplemental Table E3). Therefore, estimates of the treatment-related increase in the CVD AMR_30_ also varied substantially between the different geographic regions and between male and female patients (Figs. 1 and 2; Table 3). Despite this, when the CVD AMR_30_ for PBT and photon RT were compared, either for all patients with HL or just for those whose CTV extended below the LMSCA, the reductions for PBT compared with photon RT were small (Table 3). However, for the approximately 30% of patients whose CTV overlapped longitudinally with the heart by *≥*40%, the reductions were more substantial. For example, for U.S. background rates, the total AMR_30_ for CVD after treatment was reduced by 0.8%, from 7.0% with photon RT to 6.2% with PBT (Table 3). Additionally, the background CVD AMR_30_ for men was double that for women; thus, the predicted treatment-related AMR_30_ was also double. For example, for U.S. background rates, for patients whose CTV overlapped longitudinally with the heart by *≥*40%, the total AMR_30_ was 9.7% for photon RT and 8.6% for PBT for men versus 5.0% for photon RT and 4.4% for PBT for women, showing a benefit from PBT in both sexes for this subgroup (Fig. 2). However, for patients with <40% overlap, the AMR_30_ for photon RT and PBT was similar (men: 6.95% and 7.00%; women: 3.80% and 3.85%). The chemotherapy-related increase in the CVD AMR_30_ was substantial and, in some cases, exceeded the radiation-related AMR (Fig. 1).

**Table 3:** Predicted 30-year cumulative absolute mortality risks from CVD and second cancers for all patients and subgroups after chemotherapy and either photon RT (CT + photons) or PBT (CT + PBT)

		Average cumulative absolute mortality risk (%)(range)							
		United States		Western Europe†		Eastern Europe†		Japan	
Disease	Patient group*	CT + photons	CT + PBT	CT + photons	CT + PBT	CT + photons	CT + PBT	CT + photons	CT + PBT
**CVD**	All patients	5.6 (0.9-27.2)	5.5 (0.9-27.5)	3.2 (0.3-27.8)	3.1 (0.3-28.0)	9.7 (1.0-48.0)	9.4 (1.0-48.4)	2.7 (0.3-22.0)	2.6 (0.3-21.9)
	<40%‡	5.3	5.3	3.1	3.1	9.2	9.2	2.5	2.5
	≥40%‡	7.0	6.2	3.8	3.5	11.1	10.3	3.0	2.8
	Above LMSCA§	4.7	4.9	2.8	2.9	8.5	8.7	2.3	2.4
	Below LMSCA§	5.9	5.7	3.4	3.3	9.9	9.7	2.7	2.6
**Lung cancer**	All patients	2.4 (0.2-11.6)	2.0 (0.2-10.4)	2.4 (0.1-13.0)	2.0 (0.1-11.2)	2.7 (0.1-13.3)	2.2 (0.1-11.4)	1.6 (0.1-13.0)	1.3 (0.1-12.2)
	No axilla	2.3	1.9	2.3	1.9	2.4	2.0	1.4	1.2
	Axilla	2.7	2.1	2.7	2.2	3.0	2.3	1.8	1.4
**Breast cancer**║	All women	0.7 (0.2-1.4)	0.7 (0.2-1.3)	0.7 (0.2-1.5)	0.7 (0.2-1.5)	0.8 (0.3-1.6)	0.8 (0.3-1.6)	0.6 (0.2-1.0)	0.5 (0.2-1.0)
	No axilla	0.7	0.7	0.7	0.7	0.8	0.8	0.6	0.5
	Axilla	0.7	0.7	0.8	0.7	0.9	0.8	0.6	0.5
**Esophageal cancer**	All patients	0.2 (0.0-1.4)	0.2 (0.0-1.6)	0.3 (0.0-1.5)	0.3 (0.0-1.8)	0.2 (0.0-1.3)	0.2 (0.0-1.2)	0.3 (0.0-2.1)	0.3 (0.0-2.6)

*Abbreviations:* CT = chemotherapy; CTV = clinical target volume; CVD = cardiovascular disease; LMSCA = left main stem coronary artery; PBT = proton beam therapy; RT = radiation therapy

⁎ Subgroups are directly standardized for age and sex as described in Appendix B.

† The countries comprising Western and Eastern Europe are listed in Table E1.

‡ %CTV to heart longitudinal overlap was < or ≥40%

§ CTV was above LMSCA only or extended below LMSCA

║ Female patients only (n = 50)

The background AMR_30_ for SC varied less across the 4 regions than for CVD; thus, treatment-related increases in AMR_30_ also differed less across regions. The total AMR_30_ for lung cancer was 2 to 3 times higher than the AMR_30_ for breast cancer and up to 10 times higher than that for esophageal cancer (Fig. 1). The radiation-related increase in lung cancer AMR_30_ was smaller for PBT than for photon-RT and, considering all patients with HL in conjunction with U.S. background rates, AMR_30_ was reduced by 0.4% with PBT from 2.4% to 2.0% (Fig. 1). When patients with treated axillae were considered separately, the absolute difference in lung cancer AMR_30_ between PBT and photon RT was slightly larger when the axilla was irradiated than when not (0.6% vs 0.4%; Table 3), and the difference was similar for men and women (Fig. 2). The AMR_30_ for both female breast and esophageal cancers was lower than for either CVD or lung cancer, and the differences between PBT and photon RT were negligible (Fig. 1; Table 3).

### Smoking

**Fig. 3:**
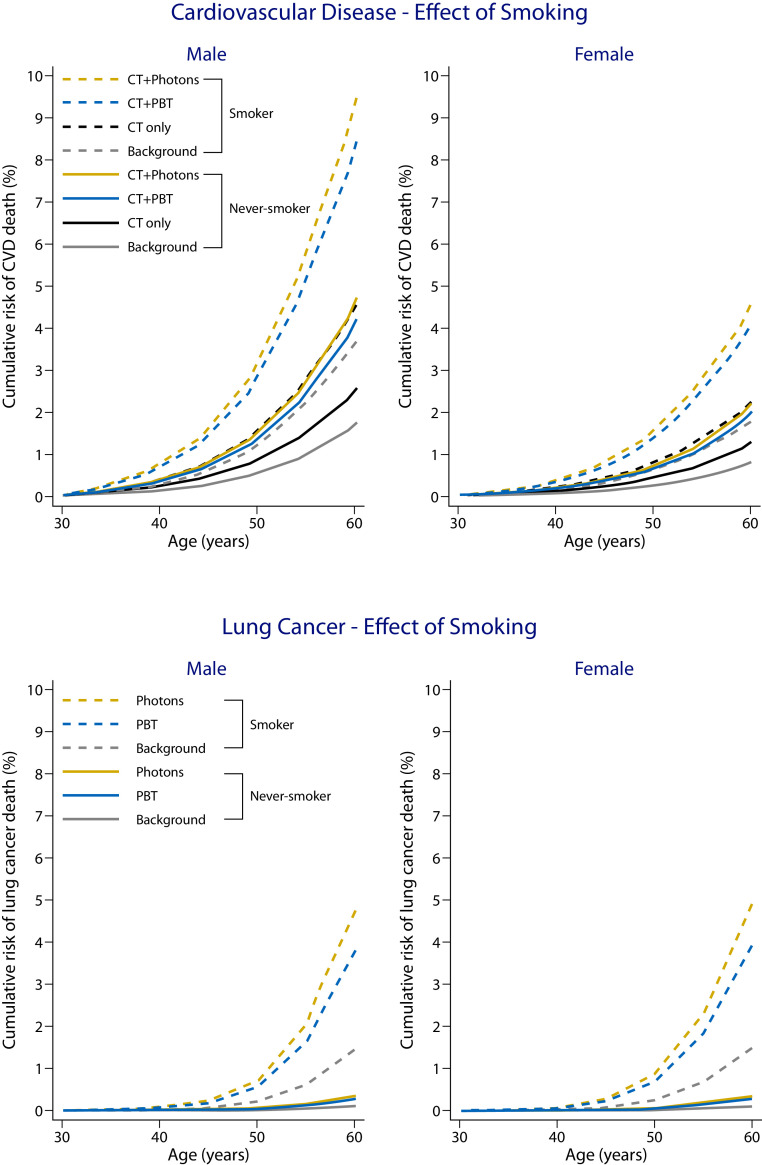
Predicted 30-year absolute mortality risks (AMR_30_) for all cardiovascular disease and lung cancer as a second primary cancer after photon radiation therapy and proton beam therapy for male and female 30-year-old current smokers and male and female 30-year-old never smokers. The background mortality was calculated using U.S. mortality rates. The chemotherapy-related risk was calculated using excess rate ratios by Van Nimwegen et al.^3^ The radiation-related AMR for cardiovascular disease was calculated assuming the average mean whole heart, left ventricle, and valve doses in patients with ≥40% clinical target volume to the heart overlap. For lung cancer, the average mean lung dose received by patients with axillary disease was used. These doses are shown in Table 2, and further details are provided in Appendices B and C.

Smoking had a strong influence on the predicted AMR_30_ for both CVD and lung cancer and on the differences between PBT and photon RT. For the U.S. background rates, the AMR_30_ for CVD for a 30-year-old male or female typical current smoker was more than double than that for a never-smoker of the same age (male: 3.7% vs 1.7%; female: 1.8% vs 0.8%), and the background AMR_30_ for lung cancer was 14 times higher in current smokers than in never-smokers (male: 4.0% vs 0.3%; female: 4.2% vs 0.3%; Fig. 3). In fact, a 30-year-old current smoker who did not receive any treatment for HL had a predicted CVD AMR_30_ almost as big as that for a never-smoker of the same age and sex who received anthracycline chemotherapy followed by RT with high cardiac doses, such as those received by patients with *≥*40% CTV-to-heart overlap.

An even stronger effect was observed for lung cancer where just the background lung cancer AMR_30_ for a current smoker was almost 10 larger than that for a never-smoker of the same age and sex who received anthracycline and high cardiac dose RT. Accordingly, compared with photon RT, PBT provided a larger absolute benefit in AMR_30_ reduction for current smokers than for never-smokers.

## Discussion

This is the first study to identify subgroups of patients who are likely to benefit from PBT versus optimal photon RT, not only dosimetrically but also by using the more clinically meaningful endpoints of predicted AMR_30_ for CVD and SC. Clinical data from a large group of patients with HL and mediastinal disease already treated with advanced PBT were used to evaluate the recent ILROG guidelines for patient selection,^19^ as well as explore possible new selection criteria. In addition, the effect of other risk factors, such as background population risk from 27 countries across 4 geographic regions, chemotherapy-related risk, and smoking, were considered for the first time.

One of the most important findings of this study is that smoking increases treatment-related AMR_30_ from CVD and lung cancer to a much larger extent than the reduction seen in these measures when PBT is used instead of photon RT. For example, just the background lung cancer AMR_30_ for a current smoker was almost 10 times larger than that for a never-smoker of the same age and sex who received anthracycline and a high cardiac RT dose. Therefore, although PBT provided a benefit compared with photon RT in AMR_30_ reduction for current smokers, smoking cessation would reduce these risks much more. Additionally, the differences in background 30-year risk between regions at times dwarfed the differences between RT modalities within the same region. For example, on average, the background AMR_30_ for CVD for the U.S. population was almost as high as the background AMR_30_ plus the additional risk from RT and chemotherapy combined for the Western European population.

These findings demonstrate that considering dosimetric factors alone is inadequate when determining PBT referral criteria, and that the magnitude of other background risk factors for CVD and cancer (eg, smoking) can be more relevant. In addition, these findings highlight the importance of active intervention regarding modifiable risk factors, in particular smoking cessation advice in young patients with HL, to reduce future CVD and lung cancer mortality risk.^26^ Frequent screening of higher-risk survivors (eg, smokers) after treatment could also reduce lung cancer mortality risk.^27^

The study shows that, for a cohort of patients selected clinically and treated with PBT, the predicted benefit of PBT is not universal and limited to certain categories of patients. This confirms the need for reliable, pragmatic selection criteria to obtain a meaningful benefit from this scarce and expensive technology. When patients were assessed as a whole group, PBT significantly reduced doses to most OARs compared with optimal photon RT, but the reductions in the overall average AMR_30_ were small. This is consistent with the findings by Rechner et al., who found small differences for all patients considered together when comparing estimated life-years lost between PBT and photon RT in DIBH.^28^ If the available alternative to PBT is a less optimal photon RT method, PBT may well provide substantial benefits for a larger proportion of patients.

For cardiac doses and CVD AMR_30_, we investigated the ILROG recommendation suggesting that patients with mediastinal disease extending below the origin of the LMSCA may benefit more from PBT.^19^ In our study, 82% of patients fell within this category and, on average, benefitted dosimetrically from PBT compared with those with disease only above the LMSCA (Table 2). The patients also benefitted somewhat in terms of CVD AMR_30_ (Table 3); thus, validating the ILROG recommendation. However, larger reductions in dose and CVD AMR_30_ were observed for the approximately 30% of patients whose CTV overlapped longitudinally with the heart by ≥40%, but there was no reduction in CVD AMR_30_ for those with a <40% overlap (Tables 2 and 3). Using U.S. background rates, the reduction in CVD AMR_30_ from PBT was 0.2% based on the ILROG-LMSCA guidelines and 0.8% based on the overlap between the RT target volume (CTV) and the heart. This more selective threshold identifies patients who will benefit more from PBT, and could help with case selection when access to PBT is limited.

The second group identified by ILROG was patients requiring RT for axillary disease, particularly young women, owing to reduced exposure of the lung and breasts from PBT. In our study, PBT reduced the lung dose and lung cancer AMR_30_ for all patients, and those whose field included the axilla had greater benefits (Tables 2 and 3). This shows a clear advantage of using PBT to reduce lung dose, even compared with BVMAT, despite the latter being effective at sparing lung tissue.^11^^,^^17^^,^^18^ Both PBT and photon RT spared the breasts, and the AMR_30_ was low for both techniques, with little additional predicted benefit with PBT. However, if optimal breast-sparing photon RT is not available, referring young women (age ≤24 years) for PBT may be appropriate, because they have a higher relative risk of breast cancer per Gy of breast dose received compared with older women (Supplemental Table E3). Of note, the AMR_30_ for lung cancer was 2 to 3 times higher than that for breast cancer and up to 10 times higher than that for esophageal cancer (Fig. 1). Contrary to popular belief that PBT should be widely used to reduce breast cancer risk, our study shows that the main mortality benefit of PBT was to reduce lung cancer AMR_30._ However, of note, our data relate to mortality risk, not incidence of breast cancer, and the conclusions may be different if incidence was the outcome rather than AMR_30_.

Our study also shows that PBT reduced the total normal tissue dose, spinal cord dose, and volumetric lung doses, such as V_5_ and V_20_ (ie, percentage volume of lung receiving at least 5 Gy and 20 Gy, respectively; Table 2; Supplemental Table E5). This is particularly beneficial for patients with relapsed disease who have already received RT because the risk of acute cord and bone marrow toxicities may lower, as well as the risk of radiation pneumonitis.^29^

The mean OAR doses for the PBT plans in our patients were comparable with those of previous studies,^11^^,^^28^^,^^30^ but our photon RT doses were lower than in other studies. For example, the average MHD, MLD, and MBD for photon RT in our study was 9.5 Gy, 7.9 Gy, and 2.5 Gy, respectively, but a recent review of 16 studies in patients with mediastinal HL reported an average MHD, MLD, and MBD of 11.4 Gy, 9.4 Gy, and 4.4 Gy, respectively.^30^ As a result, the differences between PBT and photon RT are narrower than the differences reported in the latest critical review by the Particle Therapy Cooperative Group lymphoma subcommittee.^31^ At least in part, this is because we maximized the use of partial-arc techniques; thus, avoiding the breasts and lateral parts of the lungs.^10^^,^^11^^,^^17^^,^^23^^,^^32^

An additional explanation could be the use of smaller CTV–PTV margins for photon RT, resulting in smaller target volumes for the photon RT plans compared with PBT. These margins reflect current clinical practice within our photon and proton centers, and may be smaller than those at other centers. The reduction in the magnitude of the benefit from PBT in our study highlights the importance of continually comparing technologies as they develop and improve.

## Strengths

This study is the largest study to date comparing patients with HL (n = 80) treated with optimal PBT technology using pencil beam scanning delivered in DIBH to optimal photon RT, and is the first to compare the AMR_30_ for CVD and SC. Therefore, the study provides an accurate comparison between the best available form of the 2 technologies. We use a prediction method, based on contemporary age- and sex-specific background mortality rates from 27 countries, combined with treatment-related risks from epidemiologic studies of long-term HL survivors. This is the first time that the most clinically important predicted mortality risks for survivors have been combined in a model to compare PBT and photon RT.

Additionally, we predict the risk of CVD based on doses to individual cardiac substructures and arteries. This is more informative than using MHD, because there can be substantial differences between doses received by different structures due to the high conformity provided by modern photon and proton techniques.^33^ Estimating chemotherapy-related AMR_30_ for CVD alongside radiation-related risks provides a useful perspective on the relationship between the excess risks from each treatment. This is also more clinically meaningful because all patients who receive RT will first have had chemotherapy. Lastly, illustrating the large detrimental effect of smoking provides novel data for clinicians to demonstrate to their patients the importance of smoking cessation during and after treatment for HL.

## Limitations

A first limitation is that the study does not provide estimates of the absolute incidence of CVD or SC, which would be higher than mortality (AMR_30_). This is particularly relevant to CVD and breast cancer for which incidence is many times higher than mortality. Unfortunately, country-specific incidence rates by sex and age are not currently available for individual CVD for the 27 countries used in this study; thus, calculating excess incidence rates was not possible.

Second, despite recent evidence for chemotherapy-related mortality risk of second breast cancer in patients with HL,^34^^,^^35^ there is no published dose–response relationship; thus, we could not take account of this additional risk in our model. Third, the late effects from HL treatments typically do not manifest for at least a decade after completion of therapy, and continue to develop beyond 30 years from treatment.^12^^,^^36^ This latency means that directly determining the risk of late effects from patients treated recently with modern RT is not yet possible. Instead, risk predictions based on dose–response relationships derived by the best currently available follow-up studies of patients with HL treated in the past combined with individual radiation doses received by patients treated with modern RT can provide useful approximate results, and determine the potential risk reductions achievable with modern treatment approaches.^37^

Our results are based on such predictions, and there are uncertainties associated with these models, some of which can be quantified. Confidence intervals for the coefficients of the dose–response relationships are available (Supplemental Table E3), and the impact of dose–reconstruction uncertainties on them has previously been evaluated and found to be small.^38^ Additionally, as data are not available for traditional CVD risk factors, other than smoking, we cannot provide separate risks for patients with risk factors such as diabetes and elevated cholesterol levels. If such data became available, our models could be updated to demonstrate the effect of these additional risk factors. Lastly, all dose–response relationships used to predict AMR_30_ from photon RT and PBT were derived from epidemiologic studies on patients with HL treated with photon RT only. Therefore, potential proton-specific effects (eg, those caused by uncertainties in the relative biologic effectiveness of protons^39^) could not be taken into account.

## Conclusions

Patients with supradiaphragmatic lymphoma who benefit the most from PBT compared with photon RT can be identified using simple and reliable criteria. Patients with low mediastinal disease (>40% CTV-to-heart longitudinal overlap) or axillary disease have a lower mortality risk from PBT, even compared with optimized photon RT, and prioritizing these individuals may be appropriate when selecting patients for PBT. Others might still receive a small dosimetric benefit with PBT, but our results suggest that this does not translate into a substantial mortality benefit.

This study also highlights the fact that, when deciding whether to refer a patient for photon RT or PBT, just considering dose comparisons is not sufficient. Background mortality risks vary considerably with age, sex, and between different geographic regions, and the effects of smoking and chemotherapy are substantial. All these factors will affect the absolute magnitude of the radiation-related risk for an individual patient. Smoking cessation should be strongly encouraged in smokers who require thoracic RT.

## Appendix Supplementary materials

Image, application 1
