# Supplementary material for: Proton Therapy in Supradiaphragmatic Lymphoma: Predicting Treatment-Related Mortality to Help Optimize Patient Selection
Source: Int J Radiat Oncol Biol Phys. 2022 Mar 15;112(4):913–25. doi: 10.1016/j.ijrobp.2021.10.151 (PMC8865523; doi:10.1016/j.ijrobp.2021.10.151)
Supplement: Supplementary file 1 [file mmc1.docx]

**Proton Therapy in Supradiaphragmatic Lymphoma:**

**Predicting Treatment-Related Mortality to Help Optimize Patient Selection**

**Supplementary Materials**

Table of contents

# Appendix A: Patient subgroups for cardiac dose comparison ……………2

# Figure E1. Anatomical distribution of the clinical target volume (CTV - yellow contour) in relation to the heart (pink contour)………………………….3

# Appendix B: Methods for predicting treatment-related absolute mortality risks (AMRs) from cardiovascular disease and second cancers………….4

i. Population background mortality risk

ii. Predicting treatment-related absolute excess mortality risk from CVD

iii. Predicting treatment-related absolute excess mortality risk from second cancers

Table E1. The countries comprising each geographical region and in parentheses the latest year that mortality rates were available for each country……….………………………………………………………………………...………..………………4

Table E2. Categorization of diseases……………………..…….….………..5

Table E3. Summary of published dose-response relationships and dose metrics used to estimate treatment-related absolute mortality risk from cardiovascular disease and second cancers for Hodgkin lymphoma patients…………………………………………………………………………………………………...….….7

# Appendix C: Method for predicting the effect of smoking on treatment-related absolute mortality risks from cardiovascular disease and second cancers………………………………………..………………………………………………….…11

Table E4. Toxicities following proton beam therapy (PBT)…….….13

Table E5. Average dose metrics for additional organs at risk and target volumes from photon-RT (Photons) and proton beam therapy (PBT)….14

References…………………………………………………………………………………….………………16

#

# Appendix A: Patient subgroups for cardiac dose comparison

The cardiac doses were compared for various subgroups of patients as described in the main manuscript and shown in Figure E1 below.


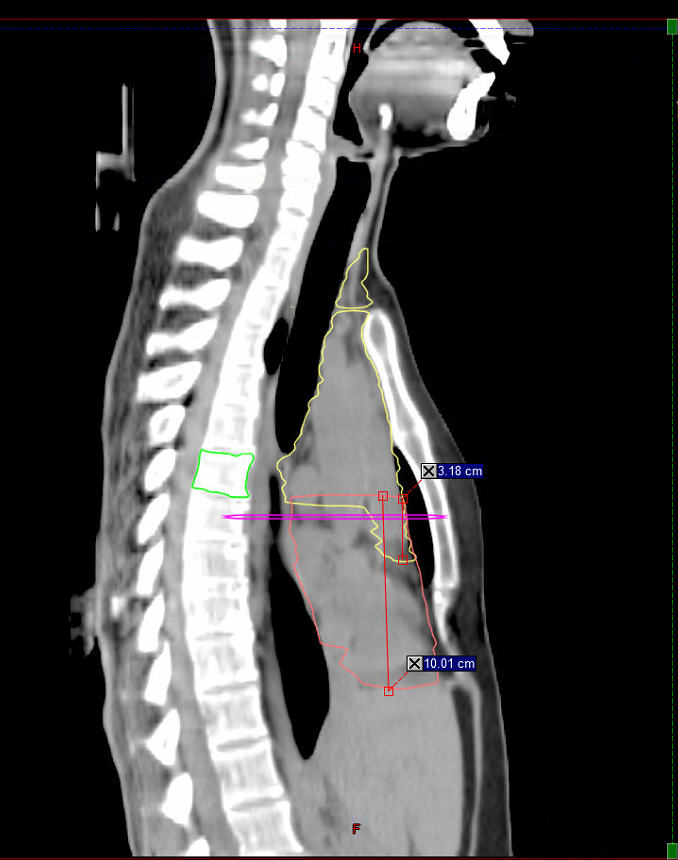


Figure E1. Anatomical distribution of the clinical target volume (CTV - yellow contour) in relation to the heart (pink contour). The %CTV to heart longitudinal overlap was estimated by dividing the overlapping length between the CTV and heart by the whole length of the heart [% CTVtoHeart = (overlapping length/length of heart) x 100, in this case 3.18/10.01 x 100 = 31.8%]. The 7^th^ thoracic vertebra can be seen in green contour as well as the level of the origin of the left main stem coronary artery (LMSCA) in magenta.

#

# Appendix B: Methods for predicting treatment-related absolute mortality risks (AMRs) from cardiovascular disease and second cancers

i Population background mortality risk

It was assumed that for any patient, their mortality rate in the absence of any effects of treatment for HL would be equal to the corresponding attained-age-and-sex-specific mortality rate in the general population of their local geographical region. For each region of interest in the study the most recent mortality rates available were used, so that our predictions would be as relevant as possible to the likely future mortality for a patient being treated currently. This is similar to the use of mortality rates that is commonly made in the construction of actuarial life tables and in calculations of life-expectancy.

Mortality rates were extracted from the World Health Organization (WHO) mortality database in 5-year attained-age groups for females and males. For each country, rates were extracted for each disease of interest for the latest year for which data were available. The HL patients included both sexes and were treated at a variety of ages. Therefore, for each of the 80 patients, the relevant attained-age-and-sex-specific rates were obtained for each of the 30 years following treatment. Rates for the United States and Japan could be calculated directly in this way, and rates for West and East Europe were derived by calculating the population-weighted average of the rates for the relevant countries, as listed in Table E1. The disease categories used, together with their respective International Statistical Classification of Diseases codes (ICD-10), are listed in Table E2. These rates will be referred to as background mortality rates.

Table E1. The countries comprising each geographical region and in parentheses the latest year that mortality rates were available for each country. *(Data extracted from WHO database 22/10/2020)*

| **Geographical Region** | **Country (year of latest mortality rates available)** |
| --- | --- |
| Western Europe | Austria (2015), Belgium (2015), Denmark (2015), Greece (2015), France (2014), Germany (2015), Ireland (2014), Italy (2015), Luxembourg (2015), Netherlands (2016), Norway (2015), Portugal (2014), Spain (2015), Sweden (2016), Switzerland (2015), United Kingdom (2013) |
| Eastern Europe | Bulgaria (2014), Croatia (2016), Czech Republic (2016), Hungary (2016), Poland (2015), Lithuania (2015), Latvia (2016) Slovakia (2014) |
| USA | United States of America (2015) |
| Japan | Japan (2015) |

Table E2. Categorization of diseases

| Disease category | ICD-10 codes |
| --- | --- |
| Cardiovascular disease (CVD) |  |
| All cardiac diseases | I01, I02.0, I05-I09, I11, I13, I20-I25, I27, I30-I52 |
| Coronary heart disease (CHD) | I20-25 |
| Valvular heart disease (VHD) | I05-I08, I09.1, I34-39 |
| Cardiomyopathy and congestive heart failure (CHF) | I01.2, I09.0, I11.0, I13.0, I13.2, I40-43, I50, I51.4-5, I51.7 |
| Other cardiac diseases | I01.0-1, I01.8-9, I02.0, I09.2-9, I11.9, I13.1, I13.9, I27, I30-33, I44-49, I51.0-3, I51.6, I51.8-9, I52 |
| Stroke | I63-69 |
| Second cancers (SC) |  |
| Breast cancer | C50 |
| Lung cancer | C34 |
| Esophageal cancer | C15 |

For each of the four regions of interest, the predicted 30-year background mortality risk for all CVD and for each of the three specific cancers of interest, taking into account the probability of death from other causes (“competing risks”), was calculated for each of the 80 patients as follows:

Let the annual death rate in the region of interest for the specific disease of interest during the n^th^ year after treatment be denoted by $r_{n}^{sp}$, and the corresponding death rate for all other causes of death be denoted by $r_{n}^{oth}$. Then the probability – or risk - of death from the specific disease of interest during year n, given that the individual has survived up to the beginning of year n, is:

$\boldsymbol{Eq.}\left( \boldsymbol{B}\boldsymbol{1} \right) \boldsymbol{p}_{\boldsymbol{n}}^{\boldsymbol{sp}}\boldsymbol{=1-}\boldsymbol{e}^{\boldsymbol{-r}_{\boldsymbol{n}}^{\boldsymbol{sp}}}$

and the probability that the individual died either of the specific disease of interest or of any other cause, given survival up to the beginning of year n, is:

$\boldsymbol{Eq.}\left( \boldsymbol{B}\boldsymbol{2} \right) \boldsymbol{p}_{\boldsymbol{n}}^{\boldsymbol{sp+oth}}\boldsymbol{=1-}\boldsymbol{e}^{\boldsymbol{-(}\boldsymbol{r}_{\boldsymbol{n}}^{\boldsymbol{sp}}\boldsymbol{+}\boldsymbol{r}_{\boldsymbol{n}}^{\boldsymbol{oth}}\boldsymbol{)}}$

while the probability that the individual survived to the end of year n, given survival up to the beginning of year n is:

$\boldsymbol{Eq.}\left( \boldsymbol{B}\boldsymbol{3} \right) \boldsymbol{q}_{\boldsymbol{n}}^{\boldsymbol{sp+oth}}\boldsymbol{=1-}\boldsymbol{p}_{\boldsymbol{n}}^{\boldsymbol{sp+oth}}$ .

Hence, for n=2,3,… the overall probability that the individual survived up to the beginning of year n is:

$\boldsymbol{Eq.}\left( \boldsymbol{B}\boldsymbol{4} \right) \boldsymbol{Q}_{\boldsymbol{n-1}}^{\boldsymbol{sp+oth}}\boldsymbol{=}\boldsymbol{Q}_{\boldsymbol{n-2}}^{\boldsymbol{sp+oth}}\boldsymbol{\times}\boldsymbol{q}_{\boldsymbol{n-1}}^{\boldsymbol{sp+oth}}$

where $\boldsymbol{Q}_{\boldsymbol{0}}^{\boldsymbol{sp+oth}}$ = 1, and the overall probability that the individual survived up to the beginning of year n and died during year n from the cause of interest is:

$\boldsymbol{Eq.}\left( \boldsymbol{B}\boldsymbol{5} \right) \boldsymbol{p\_cr}_{\boldsymbol{n}}^{\boldsymbol{sp}}\boldsymbol{=}\boldsymbol{p}_{\boldsymbol{n}}^{\boldsymbol{sp}}\boldsymbol{\times}\boldsymbol{Q}_{\boldsymbol{n-1}}^{\boldsymbol{sp+oth}}$ .

The cumulative probability that the individual dies of the cause of interest by the end of year n (i.e. the n-year cumulative mortality) is therefore:

$\boldsymbol{Eq.}\left( \boldsymbol{B}\boldsymbol{6} \right) \sum_{\boldsymbol{i=1}}^{\boldsymbol{n}} \boldsymbol{p\_cr}_{\boldsymbol{i}}^{\boldsymbol{sp}}$ .

For each year, the cumulative probability of death up to an including that year for an individual was taken to be the predicted risk of mortality up to that year. The predicted 30-year risks of mortality were then averaged over the 80 individual patients to obtain the 30-year absolute mortality risk (AMR_30_).

ii Predicting treatment-related absolute excess mortality risk from CVD

It was assumed that the characteristics of the 80 patients who received PBT for HL were a representative sample of patients receiving such treatment in each of the four geographical regions of interest. Mortality rates, including the effect of treatment, were therefore calculated for each patient and for each of the 30 years following treatment by combining the population-based mortality rates for each of the four geographical regions of interest with the patient-specific cardiac radiation doses and the dose-response relationships shown in Table E3. Dose-response relationships for the effect of radiation have been published for coronary heart disease (CHD), valvular heart disease (VHD) and congestive heart failure (CHF), and these were combined with mean heart doses (MHD), mean valve doses (MVD) and mean left ventricular doses (MLVD) respectively for each disease. For CHF separate calculations were carried out including the effect of anthracycline chemotherapy without RT as well as for the effect of combined modality treatment (i.e. chemotherapy followed by RT), using the dose-response relationship shown in Table E3.

**Table E3. Published dose-response relationships and dose metrics from studies of Hodgkin lymphoma (HL) survivors used to estimate treatment-related absolute mortality risk from cardiovascular disease and second cancers for current HL patients.**

| **Disease** | **Reference** | **Study population** | **Dose metric** | **Risk model** |
| --- | --- | --- | --- | --- |
| Coronary heart disease | *Van Nimwegen et al.*^1^ *(2016)* | 325 cases and 1,024 controls  Median age at treatment:32 y  Treated 1965-1995 | Mean Heart dose (MHD) | RR = 1 + ERR x MHD  ERR=0.200 (95% CI 0.054-0.705) if age_tx <28 y  ERR=0.088 (95% CI 0.026-0.229) if 28≤ age_tx ≤36 y  ERR=0.042 (95% CI –0.004-0.111) if age_tx >36 y |
| Valvular heart disease | *Cutter et al.*^2^ *(2015)* | 89 cases and 200 controls  All patients age at treatment <41y  Treated 1965-1995 | Summation of dose to valves (SumValve*) | RR=1 + e^(α )^ x SumValve x e^(β x SumValve)^  α = -5.02 (95% CI –9.867 to -1.915)  β = 0.075 ( 95% CI 0.007 to 0.177) |
| Congestive heart failure | *Van Nimwegen et al.*^3^ *(2017)* | 91 cases and 278 controls  Median age at treatment:28 y  Treated 1965-1995 | Mean left ventricular dose (MLVD) | RR=1+e^(-4.12)^ x MLVD x e^(0.06 x MLVD)^ +e^0.97 x danthra^  d_anthra_=1 if anthracyclines are given  d_anthra_=0 if no anthracyclines are given  CI for parameter estimates not given in ref 3. See paper for confidence intervals for RR for dose categories. |
| Stroke | De Bruin et al.^4^ (2009)  &  Maraldo et al.^5^ (2015)† | 55 cases  All patients age at treatment <51y  Treated 1965-1995 | Mean dose to the common carotid arteries (MDCCA) | RR=1+ ERR x MDCCA/ (31.85/1.286)  ERR= 2.8 (95% CI 0.6-6.4) if age_tx ≤20 y  ERR= 2.1 (95% CI 0.9-3.6) if 20< age_tx ≤30 y  ERR= 1.0 (95% CI 0.2-2.1) if 30< age_tx ≤40 y  ERR= 0.4 (95% CI – 0.3-1.3) if age_tx >40 y |
| Lung Cancer | *Travis et al.*^6^ *(2002)*  *& Gilbert et al.*^7^ *(2003)* | 222 cases and 444 controls  Treated 1965-1994 | Mean lung dose (MLD)‡ | RR=1+(ERR x MLD x 1.672)  ERR = 0.15 (95% CI: 0.06 to 0.39) |
| Breast Cancer | *Swerdlow et al.*^8^ *(2012)*  *&*  *Schaapveld et al.*^9^ *(2015)* | Cohort study of 5002 women (373 cases)  Treated 1956-2003  *&*  Cohort study including 1698 women (183 cases)  Treated 1965-2000 | Mean breast dose (MBD)‡ | RR=1+(ERR x MBD x 1.608)  ERR =0.257 if age_tx<20 y  ERR =0.097 if 20< age_tx ≤24 y  ERR =0.057 if 24< age_tx ≤30 y  ERR =0.043 if 30< age_tx ≤34 y  ERR =0.030 if age_tx>34 |
| Esophageal Cancer | *Morton et al.*^10^ *(2014)* | 36 cases and 71 controls  Treated 1943-1992 | Mean esophageal dose (MOD)‡ | RR=1+(ERR x MOD x 1.052)  ERR= 0.10 (95%CI: 0.05 to 0.18) |

*Abbreviations*: RR: rate ratio or relative risk; ERR: excess rate ratio, or excess relative risk, per gray; CI: confidence intervals; age_tx: age at treatment in years.

* SumValve = (0.553 x AVMean) + (0.368 x MVMean) + (0.079 x TVMean), i.e., a weighted average of the mean doses to the aortic valve (AVMean), the mitral valve (MVMean) and the tricuspid valve (TVMean) based on data from Cutter at el.^2^

† Standardized incidence ratios by De Bruin et al. were used with the dose reconstruction methodology by Maraldo et al.^5^ to estimate the ERR, see Appendix B for details.

‡Adjustment factors to allow the use of mean organ dose for predictions (rather than point dose at site of second cancer development) calculated as the ratio between the mean point dose/prescribed dose from the relevant studies^6,10,11^ to mean organ dose/prescribed dose (obtained from Maraldo et al.^5^), see Appendix B for details.

No dose-response relationship was available for the “other cardiac” diseases. Therefore, the radiation-related increase was estimated by selecting one of the dose-response relationships for CHD, VHD or CHF and the corresponding cardiac dose for each component disease in the group of “other cardiac” diseases as shown in Table E2. The selection was performed under clinical guidance and was based on the disease characteristics and whether the mechanisms of radiation-induced damage was likely to be similar to one of the three main cardiac diseases. For example, for “ischemic cardiomyopathy (ICD10 I255)” the dose-response relationship for CHD was used. The mortality rates for each disease, taking into account the estimated radiation-related increase, were then added up to form the overall mortality rate for the “other cardiac” category.

There is no published dose-response relationship for stroke. Therefore an approximate dose-response relationship was constructed by considering the standardized incidence ratios (SIRs) for the risk of stroke in a cohort of HL survivors treated with mantle field RT, published by de Bruin et al.^4^, in conjunction with a retrospective dose reconstruction method for estimating mean dose to the common carotid arteries (MDCCA) as a percentage of the prescribed dose for HL patients treated with mantle field RT, published by Maraldo et al.^5^ The Maraldo et al.^5^ method was used to estimate that the average MDCCA for the individuals in the de Bruin et al.^4^ cohort was 31.85 Gy. The SIRs reported in the de Bruin et al. study were therefore divided by 31.85 in order to derive an ERR per Gy of MDCCA for the risk of stroke. Not all cases of stroke or transient ischemic attack (TIA) reported in the de Bruin study were instances of first diagnosed CVD and it was reported that prior heart disease increased the risk of stroke by a factor of 2.1 compared to having no prior heart disease. As the focus in our study was the first cardiovascular event, the above ERR/Gy was thus scaled to account for a reduced risk of stroke as the first cardiovascular event. Of the 1651 irradiated cases, 26% had prior heart disease and 74% of patients did not. Therefore, if the ERR/Gy based on the de Bruin et al study in combination with the Maraldo methods is y, then the estimated ERR/Gy for the present study is 0.74x + 2.1*0.26x = y, i.e. x=y/1.286.

The mortality rates of each of the four categories of cardiac disease and that for stroke were added together to obtain the mortality rate for all CVD taking into account the likely effects of treatment. Predictions of the 30-year cumulative absolute mortality risk (AMR_30_) for all CVD, including the likely effects of HL-treatment, were then obtained in a similar fashion to that described above for the prediction of 30-year cumulative background mortality. The difference between the predicted AMR_30_ including and excluding the effect of treatment was taken to be the treatment-related excess AMR_30_ from CVD. These calculations were repeated for each of the four geographic regions of interest.

The average cumulative AMR_30_ for the group “All patients” shown in Table 3 in the main manuscript were calculated as the average of the 80 patient-specific risks_._ These were calculated as described above, using patient-specific age-and- sex-specific background mortality rates and patient-specific cardiac, lung and breast doses. To predict the average cumulative AMR_30_ for the subgroups (e.g. “≥ or <40% CTVtoHeart overlap” and “above or below LMSCA”) the patient-specific background mortality rates were used once again. However, to eliminate differences in the distribution of age and sex between the different subgroups, the results for the subgroups in Table 3 were obtained by assigning the average mean dose for each subgroup (as seen in Table 2) to all 80 patients and then calculating the average.

iii Predicting treatment-related absolute excess mortality risk from second cancers

Predictions of the AMR_30_ for each cancer of interest (lung, breast and esophagus), taking into account the effect of radiotherapy, were obtained in a similar fashion to that described above for CVD. The dose-response relationships shown in Table E3 were used combined with the patient-specific radiation doses to the lung, breast and esophagus for all 80 patients. As with CVD, the difference between the predicted AMR_30_ including and excluding the effect of radiotherapy was taken to be the radiotherapy-related excess AMR for each second cancer. These calculations were repeated for each of the four geographic regions of interest. The same methodology as described for CVD was used to calculate AMR_30_ for “All” and “Axilla or No Axilla” patient groups as seen in Table 3 in the main manuscript.

The published case-control studies that produced the dose-response relationships utilized the point dose at the site of the second cancer as the dose metric for exposure. However, point dose is not the most appropriate measure for predicting future risk, as it is not known in advance exactly where a future cancer may develop. Therefore, we used published dosimetry data^6,10,11^ to define the ratio between the mean dose at the point the cancer developed to the mean organ dose (estimated as a percentage of prescribed dose using the dose reconstruction method by Maraldo et al.^5^) to derive adjustment factors for the ERR per Gy used for the predictions. The adjustment factors for each type of second primary cancer are shown in Table E3.

# Appendix C: Method for predicting the effect of smoking on treatment-related absolute mortality risks from cardiovascular disease and second cancers

To estimate age-and-sex-specific mortality rates for non-smokers and smokers for a specific disease, the mortality rates for that disease in the entire US population were used, as provided by WHO, combined with estimates of the proportions of the population who are current smokers, ex-smokers or never-smokers and the relevant mortality rate ratios (RRs). These were obtained for CVD and lung cancer from the U.S. Surgeon General’s 2014 Report.^12^

In order to calculate the background mortality rates for non-smokers the following formula was used:

Eq. (C1) R_n_=R_T_/[(p_s_ x RR_s_) + (p_e_ x RR_e_) + p_n_]

where:

- R_n_: Death rate in non-smokers for the disease of interest
- R_T_: WHO age-and sex-specific death rate in the total population for the disease of interest
- p_s_: Proportion of population who are current smokers, subdivided by age and sex (Table 13.4 of the U.S. Surgeon General’s 2014 Report^12^).
- RR_s_: Mortality rate ratio for the specific disease in current smokers compared with never-smokers (Table 12.3 of the U.S. Surgeon General’s 2014 Report^12^). Current smokers were those who answered ‘yes’ in questionnaires to questions such as: ‘Do you smoke cigarettes now?’.
- p_e_: Proportion of population who are ex-smokers, split by age and sex (rates of quitting from Table 13.9 of the U.S. Surgeon General’s 2014 Report^12^ multiplied by the prevalence from the same report.)
- RR_e_: Mortality rate ratio for the specific disease in ex-smokers (Table 12.3 of the U.S. Surgeon General’s 2014 Report^12^).
- p_n_: Proportion of the population who are never-smokers, subdivided by age and sex (equal to 1- p_s_ - p_e_).

In order to calculate the background mortality rates for current smokers, R_n_ was multiplied by RR_s_. After the background mortality rates for current smokers and never-smokers had been calculated, the risk prediction methodology, as described above, was used to predict treatment-related AMR for CVD and lung cancer for a 30-year-old American male and female smoker and never-smoker HL patient for each year following treatment.

Table E4. Toxicities following proton radiotherapy

| **Acute toxicity** | **Number of patients**  **(n=80)** | **(%)** |
| --- | --- | --- |
| Dysphagia grade 0 | 21 | 26% |
| Dysphagia grade 1 | 52 | 65% |
| Dysphagia grade 2gr.2 | 7 | 9% |
| Leucopenia grade 0 | 73 | 91% |
| Leucopenia grade 1 | 3 | 4% |
| Leucopenia grade 2 | 4 | 5% |
| Radiodermatitis grade 0 | 43 | 54% |
| Radiodermatitis grade 1 | 36 | 45% |
| Radiodermatitis grade 2 | 1 | 1% |
| Lung toxicity grade 0 | 78 | 98% |
| Lung toxicity grade 1 | 2 | 2% |
| Xerostomia grade 0* | 23 | 77% |
| Xerostomia grade 1* | 7 | 23% |
| Mucositis grade 0* | 20 | 67% |
| Mucositis grade 1* | 9 | 30% |
| Mucositis grade 2* | 1 | 3% |
| *only relevant for those patients who had neck irradiation (n=30) |  |  |

Table E5. Average dose metrics for additional organs at risk and target volumes from photon-RT (Photons) and proton beam therapy (PBT)

| **Organ at risk** | | **Patient group** | **Dose metric** | **Photons (Gy)**  average (range) | | **PBT (GyE*)**  average (range) | | **Absolute difference (Gy)**  average (range) | **p-value for absolute difference** | | | p-value for difference between patient subgroups | |
| --- | --- | --- | --- | --- | --- | --- | --- | --- | --- | --- | --- | --- | --- |
| **Whole heart** | |  |  |  | |  | |  |  | | |  | |
|  |  | Above T7 ^†^ | D_mean_ | 5.5 (0.6, 12.2) | | 6.4 (1.0, 14.3) | | +0.9 (-2.7, 5.8) | p=0.08 | | p<0.001 | |  |
|  |  | Below T7 ^†^ | D_mean_ | 12.3 (3.7, 26.1) | | 10.6 (3.5, 20.7) | | -1.7 (-12.5, 3.3) | p<0.001 | |  |  |  |
| **Left ventricle** | |  |  |  | |  | |  |  | | |  | |
|  |  | Above T7 ^†^ | D_mean_ | 2.2 (0.2, 7.9) | | 1.5 (0.1, 9.0) | | -0.7 (-4.4, 1.7) | p=0.26 | | p<0.01 | |  |
|  |  | Below T7 ^†^ | D_mean_ | 7.6 (0.9, 23.9) | | 4.2 (0.0, 14.4) | | -3.3 (-18.0, 3.9) | p<0.001 | |  |  |  |
| **Heart valves ^‡^** | |  |  |  | |  | |  |  | |  | |  |
|  |  | Above T7^†^ | D_mean_ | 9.3 (0.4, 23.9) | | 9.4 (0.1, 29.2) | | +0.1 (-9.8, 5.2) | p=0.94 | | p<0.001 | |  |
|  |  | Below T7^†^ | D_mean_ | 17.4 (3.0, 28.3) | | 12.8 (1.0, 27.2) | | -4.6 (-19.5, 8.3) | p<0.001 | |  |  |  |
| **Whole heart** | | All | V_15_ (%) | 28.9 (0.0, 97.7) | 27.1 (2.0, 66.5) | | -1.8 (-59.4, 13.7) | | p=0.21 |  | | | |
|  | |  | V_30_ (%) | 4.7 (0.0, 19.8) | 13.9 (0.0, 48.4) | | +9.3 (0.0, 33.8) | | p<0.001 |  | | | |
| **Cardiac Substructures** All | | |  |  |  | |  | |  | |  | | |
| LAD | |  | D_mean_ | 10.3 (0.4, 30.3) | 11.4 (0.0, 31.3) | | +1.2 (-29.8, 23.7) | | p=0.35 | |  | | |
| L C coronary artery | |  | D_mean_ | 21.5 (0.8, 31.2) | 20.9 (0.2, 31.8) | | -0.6 (-21.1, 14.7) | | p=0.45 | |  | | |
| R coronary artery | |  | D_mean_ | 12.8 (0.4, 30.5) | 15.4 (0.0, 31.3) | | +2.6 (-12.8, 11.0) | | p<0.001 | |  | | |
| Aortic Valve | |  | D_mean_ | 19.1 (0.5, 30.3) | 18.2 (0.1, 31.2) | | -0.9 (-25.2, 12.4) | | p=0.22 | |  | | |
| Pulmonary Valve | |  | D_mean_ | 22.4 (0.5, 31.9) | 26.2 (0.1, 31.8) | | +3.8 (-14.0, 21.3) | | p<0.001 | |  | | |
| Mitral Valve | |  | D_mean_ | 8.1 (0.4, 28.2) | 2.7 (0.0, 27.6) | | -5.5 (-23.9, 12.1) | | p<0.001 | |  | | |
| Tricuspid Valve | |  | D_mean_ | 6.2 (0.1, 29.6) | 4.4 (0.0, 31.6) | | -1.8 (-25.7, 12.2) | | p=0.01 | |  | | |
| Right Ventricle | |  | D_mean_ | 7.7 (0.2, 28.0) | 7.8 (0.0, 26.7) | | +0.1 (-23.6, 23.2) | | p=0.94 | |  | | |
| L atrium | |  | D_mean_ | 13.9 (0.7, 29.7) | 8.4 (0.1, 29.8) | | -5.5 (-22.4, 9.3) | | p<0.001 | |  | | |
| R atrium | |  | D_mean_ | 10.4 (0.6, 28.4) | 9.8 (0.1, 31.4) | | +0.6 (-14.9, 6.6) | | p=0.27 | |  | | |
| **Lungs** | | All | V_5_ (%) | 41.3 (20.2, 75.9) | 25.2 (11.4, 44.0) | | -16.1 (-43.0, -4.1) | | p<0.001 | |  | | |
|  |  |  | V_20_ (%) | 16.1 (4.5, 33.3) | 14.4 (5.7, 26.8) | | -1.6 (-16.0, 9.2) | | p<0.001 | |  | | |
| **Breast** | | Females | V_4_ (%) | 14.9 (1.1, 47.1) | 9.4 (1.1, 25.8) | | -5.5 (-31.3, 7.4) | | p<0.001 | |  | | |
|  |  |  | V_10_ (%) | 7.1 (0.0, 31.4) | 6.6 (0.5, 19.1) | | -0.5 (-20.0, 7.1) | | p=0.43 | |  | | |
|  |  |  | V_20_ (%) | 2.8 (0.0, 23.1) | 3.9 (0.0, 26.8) | | +1.1 (-2.5, 4.1) | | p<0.001 | |  | | |
| **Larynx** | | All | D_mean_ | 13.7 (0.0, 29.0) | 11.9 (0.0, 30.0) | | -1.8 (-17.3, 5.3) | | p<0.001 | |  | | |
| **Thyroid** | | All | D_mean_ | 23.2 (0.0, 31.3) | 24.4 (0.0, 31.3) | | 1.2 (-27.7, 30.3) | | p=0.05 | |  | | |
| **Spinal Cord** | | All | D_max_ | 28.4 (22.0, 32.2) | 11.0 (0.0, 29.3) | | -17.4 (-31.9, 0.6) | | p<0.001 | |  | | |
| **PTV** | | All | PTV_Volume_ (cc) | 1072 (950, 1195) | 1637 (1515, 1760) | | +565 (161, 1136) | | p<0.001 | |  | | |
|  |  |  | V95% | 96.6 (90.4, 100.0) | 99.4 (96.8, 100.0) | | +2.9 (-5.1, 4.6) | | p=0.12 | |  | | |

Abbreviations: RT= radiotherapy, Gy = Gray, T7: 7^th^ thoracic level, D_mean_ = mean organ dose, V_x_(%) the % volume receiving X Gy of radiation dose, LAD = left anterior descending artery, L = left, C = circumflex, R = right, PTV = planning target volume, cc = cubic centimeters, V95% = volume receiving 95% of the prescribed dose

** GyE is Gy equivalent of relative biological effectiveness of 1.1 with proton beam therapy*

† The CTV extended above/at or below T7

‡ SumValve = (0.553 x AVMean) + (0.368 x MVMean) + (0.079 x TVMean), i.e., a weighted average of the mean doses to the aortic valve (AVMean), the mitral valve (MVMean) and the tricuspid valve (TVMean) based on data from Cutter at el.^2^

# References

1. van Nimwegen FA, Schaapveld M, Cutter DJ, et al. Radiation Dose-Response Relationship for Risk of Coronary Heart Disease in Survivors of Hodgkin Lymphoma. *J Clin Oncol*. 2015;34(3):235-243. doi:10.1200/JCO.2015.63.4444

2. Cutter DJ, Schaapveld M, Darby SC, et al. Risk of Valvular Heart Disease After Treatment for Hodgkin Lymphoma. *JNCI J Natl Cancer Inst*. 2015;107(4):djv008. doi:10.1093/jnci/djv008

3. Van Nimwegen FA, Ntentas G, Darby SC, et al. Risk of heart failure in survivors of Hodgkin lymphoma: Effects of cardiac exposure to radiation and anthracyclines. *Blood*. 2017;129(16):2257-2265. doi:10.1182/blood-2016-09-740332

4. De Bruin ML, Dorresteijn LDA, Van’t Veer MB, et al. Increased risk of stroke and transient ischemic attack in 5-year survivors of hodgkin lymphoma. *J Natl Cancer Inst*. 2009;101(13):928-937. doi:10.1093/jnci/djp147

5. Maraldo M V, Lundemann M, Vogelius IR, et al. A new method to estimate doses to the normal tissues after past extended and involved field radiotherapy for Hodgkin lymphoma. *Radiother Oncol*. 2015;114(2):206-211. doi:10.1016/j.radonc.2015.01.008

6. Travis LB, Gospodarowicz M, Curtis RE, et al. Lung cancer following chemotherapy and radiotherapy for Hodgkin’s disease. *J Natl Cancer Inst*. 2002;94(3):182-192. doi:10.1093/jnci/94.3.182

7. Gilbert AES, Stovall M, Gospodarowicz M, et al. Lung Cancer after Treatment for Hodgkin’s Disease : Focus on Radiation Effects. *Radiat Res*. 2003;159(2):161-173.

8. Swerdlow AJ, Cooke R, Bates A, et al. Breast cancer risk after supradiaphragmatic radiotherapy for Hodgkin’s lymphoma in England and Wales: a National Cohort Study. *J Clin Oncol*. 2012;30(22):2745-2752. doi:10.1200/JCO.2011.38.8835

9. Schaapveld M, Aleman BMP, van Eggermond AM, et al. Second Cancer Risk Up to 40 Years after Treatment for Hodgkin’s Lymphoma. *N Engl J Med*. 2015;373(26):2499-2511. doi:10.1056/NEJMoa1505949

10. Morton LM, Gilbert ES, Stovall M VLF. Risk of esophageal cancer following radiotherapy for Hodgkin lymphoma. *Haematologica*. 2014;(Table 1):193-196. doi:10.3324/haematol.2014.108258

11. Travis LB, Hill DA, Dores GM, et al. BReast cancer following radiotherapy and chemotherapy among young women with hodgkin disease. *Jama*. 2003;290(4):465-475. doi:10.1001/jama.290.4.465

12. Newcomb PA, Carbone PP. The Health Consequences of Smoking —50 Years of Progress. *US Dep Heal Hum Serv*. 2014. doi:10.1016/S0025-7125(16)30355-8
